# Dalpiciclib plus aromatase inhibitor versus neoadjuvant chemotherapy for ER-positive, HER2-negative breast cancer

**DOI:** 10.3389/fonc.2025.1566146

**Published:** 2025-04-30

**Authors:** Haoqi Wang, Guoyu Zhang, Wei Gao, Sainan Li, Shan Yang, Xi Zhang, Jiaxing Wang, Chunxiao Li, Shan Gao, Cuizhi Geng

**Affiliations:** ^1^ Breast Center, The Fourth Hospital of Hebei Medical University, Shijiazhuang, Hebei, China; ^2^ Hebei Key Laboratory of Breast Cancer Molecular Medicine, The Fourth Hospital of Hebei Medical University, Shijiazhuang, Hebei, China; ^3^ Department of Breast Surgery, Handan Central Hospital, Handan, Hebei, China; ^4^ Gland Surgery, The Hebei Province People’s Hospital, Shijiazhuang, Hebei, China

**Keywords:** hormone receptor positive/human epidermal growth factor receptor 2-negative breast cancer, dalpiciclib, neoadjuvant treatment, efficiency, safety

## Abstract

**Background:**

The combination of cyclin-dependent kinases 4 and 6 inhibitors (CDK4/6i) with endocrine therapy (ET) has emerged as an effective alternative to neoadjuvant chemotherapy (NCT) for patients with hormone receptor-positive (HR+) and human epidermal growth factor receptor 2 (HER2) -negative breast cancer (BC). This single-center study retrospectively evaluated the efficacy and safety of dalpiciclib combined with an aromatase inhibitor (AI) compared to NCT.

**Methods:**

The clinicopathological data and treatment details of patients with HR+ HER2 negative BC who underwent either neoadjuvant endocrine therapy (NET) or NCT were collected from the Fourth Hospital of Hebei Medical University. The primary endpoint of the study was the Residual Cancer Burden (RCB), while secondary endpoints included breast pathological complete response (bpCR), clinical response rates (ORR), proliferation markers, and safety profiles.

**Results:**

Between May 2022 and June 2023, a total of 36 patients were treated with dalpiciclib plus AI, while 137 patients received NCT for the final analysis. Prior to propensity score matching (PSM), the rates of RCB 0 were 0% in the NET group and 7.3% in the NCT group (*p*=0.205). The rates of bpCR were 2.8% for the NET group and 13.1% for the NCT group (*p*=0.142). The ORR was comparable between the two groups (*p*=0.397), as were the rates of BCS (*p*=0.608). Both treatment groups demonstrated significant reductions in Ki-67 levels, although the extent of reduction was similar (*p*=0.174). Notably, a Ki-67 level of ≤ 10% post-treatment was more prevalent in the NET group (*p*<0.0001). Only two patients in the NET group achieved a Preoperative Endocrine Prognostic Index (PEPI) 0 score. The incidence of grade 3–4 toxicities was significantly higher in the NCT group compared to the NET group (*p*<0.05). Following PSM, patients treated with dalpiciclib plus AI exhibited comparable clinical responses and a safety advantage relative to those receiving NCT.

**Conclusion:**

This study indicates that the combination of dalpiciclib and AI elicits comparable responses and demonstrates improved safety profiles when compared to NCT in patients with HR+ HER2 negative breast tumors. Furthermore, RCB and pCR may not serve as optimal endpoints for evaluating the efficacy of CDK4/6i-based NET.

## Introduction

Neoadjuvant systemic treatment represents a pivotal therapeutic approach for patients with breast cancer (BC) in clinical practice. While chemotherapy (CT) remains the predominant option for various molecular subtypes in neoadjuvant settings, the efficacy of endocrine therapy (ET) has led to a series of investigations into neoadjuvant endocrinotherapy (NET) for patients with hormone receptor -positive (HR+) and human epidermal growth factor receptor 2 (HER2) negative tumors (1–3). Despite demonstrating comparable efficacy and significantly reduced toxicity, the curative potential of conventional ET, even when combined with ovarian function suppression (OFS), is limited in comparison to neoadjuvant chemotherapy (NCT) (4–7).

CDK4/6 inhibitors (CDK4/6is) function by inhibiting cell proliferation through the inactivation of CDK4/6, thereby facilitating the transition from the G1 phase to the S phase of the cell cycle (8). These inhibitors, in conjunction with ET, are currently utilized as a rescue strategy for advanced BC (ABC) (9–11) and as intensive adjuvant therapy for early breast cancer (EBC) (12) in HR+ and HER2 negative populations. Although findings are inconsistent, several studies have explored the efficacy of ET combined with CDK4/6is-specifically palbociclib, abemaciclib, and ribociclib-in the neoadjuvant setting since 2016 (13–19). Preliminary data indicate that the combination of CDK4/6is with ET may be a promising option for neoadjuvant therapy (NAT), given its favorable safety profile and comparable efficacy in treating pre- and postmenopausal ER+ HER2 negative EBC (1, 20). However, there is a paucity of prospective trials directly comparing the effectiveness of CDK4/6is combined with ET against NCT, with no significant advantage in pathological response observed for this regimen over NCT (1, 2). Consequently, due to the numerous unresolved questions (21), NET is not widely adopted for patients with HR+ HER2 negative BC, whether or not CDK4/6is are included (22, 23).

Dalpiciclib, a novel oral small-molecule CDK4/6i, was initially developed in China (24). Noteworthy phase III trials, DAWNA-1 and DAWNA-2,demonstrated a significant improvement in progression-free survival (PFS) with dalpiciclib in combination with fulvestrant or an aromatase inhibitor (AI) compared to placebo plus fulvestrant or AI (DAWNA-1:15.7 months vs. 7.2 months, hazard ratio (HR) 0.42, *p*<0.0001; DAWNA-2: 30.6 months vs.18.2 months, HR 0.51, *p*<0.0001) in patients with pretreated HR+ HER2 negative ABC (25, 26). A phase II clinical trial (CTR20210652) is currently ongoing to evaluate the efficacy of dalpiciclib in combination with ET in an intensive adjuvant setting for HR+ HER2 negative EBC with a high risk of recurrence. To date, there is no evidence directly comparing dalpiciclib-based NET to NCT.

This retrospective study examined the real-world clinical application of dalpiciclib in combination with ET within the neoadjuvant setting, comparing its efficacy and safety to that of NCT. Changes in Ki-67, a predictive marker for responsiveness to both NET and NCT, were evaluated post-neoadjuvant therapy in both cohorts. The preoperative endocrine prognostic index (PEPI) score, which is derived from Ki-67, serves as an additional prognostic indicator (27). Previous studies have demonstrated that the PEPI score correlates with long-term outcomes (28) and is widely utilized to assess the response to NET (1, 28). Consequently, we assessed the PEPI status of patients following NET to estimate treatment efficacy. These findings will contribute further evidence regarding the feasibility of combining CDK4/6i with ET in the NET context and will provide valuable guidance for clinical practice.

## Materials and methods

### Patients

A consecutive series of patients diagnosed with BC who received either NET or NCT at the Fourth Hospital of Hebei Medical University from May 2022 to June 2023 were included in this study. The inclusion criteria were as follows: 1) Pathologically confirmed unilateral invasive breast cancer; 2) Pathologically verified HR- and HER2 negative status; 3) Staged at II-III without invasion of internal mammary or supraclavicular lymph nodes; 4) Administration of dalpiciclib in combination with AI for postmenopausal women or concurrent treatment with gonadotropin-releasing hormone agonists (GnRHa) for premenopausal individuals in the NET setting; 5) Completion of the full course of NET (4–6 cycles/month) and NCT (6–8 cycles), followed by radical surgery within 1–4 weeks; 6) Imaging responses were evaluated every two cycles using ultrasound and magnetic resonance imaging (MRI); 7) Sufficient clinicopathological data were available.

### Clinicopathologic characteristics and variable definitions

Baseline data were collected from eligible patients, including age, menstrual status, histological type, tumor size, axillary lymph node staging, TNM stage, HR status, HER2 expression, Ki-67 index, NAT regimens, surgical methods, Miller-Payne (MP) classification (29), and residual cancer burden (RCB) status.

The expression levels of estrogen receptor (ER), progesterone receptor (PR), HER2, and Ki-67 were evaluated using immunohistochemical (IHC) staining techniques. HR positivity was defined as ER and/or PR expression levels of ≥ 1% (30). ER expression was categorized into three distinct groups: 1-10%, 11-50%, and > 50%. HER2 negativity was defined as: 0, 1+, or 2+ with fluorescence in situ hybridization (FISH) negativity, where 1+ or 2+ with FISH negativity was defined as HER2 low expression. The Luminal A subtype was characterized by ER positivity, PR expression > 20%, HER2 negativity, and Ki-67<14%, while the Luminal B subtype was defined as ER positive, PR ≤ 20%, HER2 negative, and Ki-67 ≥ 14% (14).

In accordance with the specific criteria of the MP classification, patients who attained MP scores of 4 to 5 were categorized as ‘well-responsive.’

### Assessment and endpoints

Imaging responses of the breast and the associated lymph nodes in the drainage area were evaluated at baseline, after every two cycles of NAT, and prior to surgery using breast enhanced MRI.

The primary endpoint of the study was the RCB. Secondary endpoints included the objective response rate (ORR), pathological complete response (pCR) in the breast (bpCR; ypT0/is), the status of ‘well-responsive’, the rate of breast-conserving surgery (BCS), changes in Ki-67 expression post-surgery, the PEPI score in the NET setting, and safety assessments. The ORR was determined as the proportion of patients achieving complete response (CR) and partial response (PR) prior to surgery, in accordance with the RECIST 1.1 criteria. bpCR was defined as a Miller-Payne (MP) grading of 5. Changes in Ki-67 expression during NAT were calculated as the baseline value obtained from needle biopsy minus the value obtained from postoperative pathology. The PEPI score was computed as the sum of surgical items outlined in **Table 1**. Safety was assessed using the Common Terminology Criteria for Adverse Events (CTCAE) version 5.0.

**Table 1:** The preoperative endocrine prognostic index.

Surgical factors		Points
Pathological tumor size	PT1-2	0
	PT3-4	3
Pathological Node Invasion	Negative	0
	Positive	3
Ki-67 Index	0-2.7%	0
	2.8-19.7%	1
	19.8-53.1%	2
	>53.1%	3
ER Allred score^a^	0-2	3
	3-8	0
PEPI score		Toatl score

^a^Allred Scores for Estrogen and Progesterone Receptor Assessment.

### Statistical analysis

Statistical analyses were conducted using SPSS version 26.0. The Chi-square test or Fisher’s exact test was employed to assess differences in qualitative data. Quantitative data that conformed to a normal distribution were expressed as mean ± standard deviation, with the t-test utilized for intergroup comparisons. For quantitative data that did not follow a normal distribution, results were presented as median and interquartile range [M (Q3-Q1)], and the Mann-Whitney U test was used for comparisons between groups. The Wilcoxon Z test was applied for intragroup comparisons before and after treatment. Propensity score matching (PSM) was performed to adjust for confounding variables at a 1:2 ratio using R software (version 4.3.5). The PSM was validated using standardized mean differences (SMDs). Sensitivity analyses were conducted to test robustness. The efficacy and safety of the two treatment groups were analyzed both pre- and post-matching. Statistical significance was defined as *P*<0.05 for all analyses.

## Results

### Demographic characteristics

Between May 2022 and June 2023, a total of 173 eligible patients were included in the final analysis, consisting of 36 patients who received dalpiciclib in combination with an AI (letrozole: 31 patients, anastrozole: 2 patients, or exemestane: 3 patients) and 137 patients who underwent NCT. The initial dosage of dalpiciclib was 150 mg administered orally once daily for three weeks, followed by a one-week treatment interruption. In the NCT cohort, the majority of patients (102, 74.5%) received four cycles of anthracycline plus cyclophosphamide, followed by four cycles of taxane (AC*4-T*4, every three weeks per cycle), while a smaller proportion of patients (35, 25.5%) were treated with six cycles of taxane plus anthracycline (TA*6), also on a three-week cycle.

Baseline characteristics are summarized in **Table 2**. The median age of patients in the NET group was 57 years, in contrast to 51 years in the NCT group (*p* = 0.001). A significantly higher proportion of patients in the NET group were over 50 years of age (88.9% vs. 44.5%, *p* < 0.0001) and were postmenopausal (80.6% vs. 40.9%, *p* < 0.0001). Additionally, a greater prevalence of patients with Luminal A tumors was observed in the NET group compared to the NCT group (38.9% vs. 14.6%, *p* = 0.001). No statistically significant differences were found between the two groups regarding primary tumor size, lymph node involvement, TNM stage, histological type, or HR and HER2 status (*p* > 0.05). Following NAT, 30.5% of patients in the NET group and 26.3% in the NCT group underwent BCS, with no significant differences noted (*p* = 0.608).

**Table 2:** Baseline characteristics of patients in both groups before PSM.

	Dalpiciclib+AI (36)	NCT^a^ (137)	*P*
**Median age**	57	51	**0.001**
**Age, n (%)**			**<0.0001**
≤50	4(11.1%)	76(55.5%)	
>50	32 (88.9%)	61(44.5%)	
**Menstrual status, n (%)**			**<0.0001**
premenopausal	7 (19.4%)	81(59.1%)	
postmenopausal	29 (80.6%)	56(40.9%)	
**Histological type, n (%)**			0.88
IDC**^b^**	33(91.6%)	122(89.1%)	
others	3(8.4%)	15(10.9%)	
**T stage, n (%)**			0.629
T1	14(38.9%)	53(38.7%)	
T2	17 (47.2%)	66(48.2%)	
T3	3 (8.3%)	5(3.6%)	
T4	2 (5.6%)	13(9.5%)	
**N stage, n (%)**			0.54
**N0**	2 (5.6%)	8(5.9%)	
**N1**	27 (75.0%)	115(83.9%)	
**N2**	3 (8.3%)	7(5.1%)	
**N3**	4(11.1%)	7(5.1%)	
**TNM stage^c^, n (%)**			0.196
II	25 (69.4%)	109(79.6%)	
III	11 (30.6%)	28(20.4%)	
**ER^d^, n (%)**			0.115
1-10%	0(0%)	3(2.2%)	
11-50%	0(0%)	6(4.4%)	
>50%	36(100%)	6(4.4%)	
**PR^e^, n (%)**			0.418
Positive	33(91.7%)	116(84.7%)	
Negative	3(8.3%)	21(15.3%)	
**HER2** ^f^ **, n (%)**			0.453
0-expression	8(22.2%)	39(28.5%)	
Low-expression	28(77.8%)	98(71.5%)	
**Ki-67, n (%)**			**0.001**
**≤30%**	33(91.6%)	86(62.8%)	
**>30%**	3(8.4%)	51(37.2%)	
**Subtype, n (%)**			**0.001**
Luminal A	14(38.9%)	20(14.6%)	
Luminal B	22(61.1%)	20(14.6%)	

^**a**^NCT, neoadjuvant chemotherapy, ^**b**^invasive ductal carcinoma, ^**c**^based on the 8th edition of the American Joint Committee on Cancer (AJCC), ^**d**^ER, estrogen receptor, ^**e**^PR, progesterone receptor, ^**f**^HER2, human epidermal growth factor receptor 2. Bold values are statistically significant.

### Efficacy

#### Before PSM

Radiologic assessments revealed that none of the patients treated with NET achieved a CR, while 19 patients (52.8%) demonstrated a PR, resulting in an ORR of 52.8%. In contrast, the NCT group exhibited CR and PR rates of 1.5% and 59.1%, respectively, yielding an ORR of 60.6%. Statistical analysis indicated that the ORR between the two groups was not significantly different (*p* = 0.397) (**Table 3**).

**Table 3:** Clinical response before and after PSM in both groups.

	Before PSM		*P*	After PSM		*P*
	Dalpiciclib+AI (36)	NCT^a^ (137)		Dalpiciclib+AI (27)	NCT^a^ (46)	
CR^b^	0 (0%)	2 (1.5%)		0 (0%)	0 (0%)	
PR^c^	19 (52.8%)	81 (59.1%)		17 (62.9%)	21 (45.7%)	
SD^d^	17 (47.2%)	54 (39.4%)		10 (37.1%)	25 (54.3%)	
ORR^e^	19 (52.8%)	83 (60.6%)	0.397	17 (62.9%)	21 (45.7%)	0.153

^**a**^NCT, neoadjuvant chemotherapy, ^**b**^CR, complete response, ^**c**^PR, partial response, ^**d**^SD, stable disease, ^**e**^ORR, objective response rate.

No patients in the NET group achieved a RCB 0 or RCB 1. In this cohort, 17 patients (47.2%) attained RCB 2, while 19 patients (52.8%) reached RCB 3 following NET. In contrast, within the NCT group, 7.3% of patients achieved RCB 0, 10.2% achieved RCB 1, 34.3% achieved RCB 2, and 48.2% achieved RCB 3. Statistical analysis revealed no significant difference in the rates of RCB 0 between the NET and NCT groups (7.3% vs. 0%, *p* = 0.205) (**Table 4**).

**Table 4:** Residual cancer burden after surgery before and after PSM in both groups.

	Before PSM		*P*	After PSM		*P*
	Dalpiciclib+AI (36)	NCT^a^ (137)		Dalpiciclib+AI (27)	NCT^a^ (46)	
RCB^b^ 0	0 (0%)	10 (7.3%)	0.205	0 (0%)	3 (6.5%)	0.457
RCB^b^ 1-3	36 (100%)	127 (92.7%)		27 (100%)	43 (93.5%)	

^**a**^NCT, neoadjuvant chemotherapy, ^**b**^RCB, residual cancer burden.

The postoperative MP status is presented in **Table 5**. The incidence of ‘well-responsive’ cases (MP 4-5) was significantly higher in the NCT group compared to the NET group, with rates of 29.9% and 8.4%, respectively (*p* < 0.0001). However, one patient (2.8%) in the NET group achieved a bpCR, a rate that was comparable to that observed in the NCT group, which was 13.1% (*p* = 0.142).

**Table 5:** MP grading postsurgery before and after PSM in both groups.

	Before PSM		*P*	After PSM		*P*
	Dalpiciclib+AI (36)	NCT^a^ (137)		Dalpiciclib+AI (27)	NCT^a^ (46)	
**MP 1**	0(0%)	2 (1.5%)		0 (0%)	1 (2.2%)	
**MP 2**	10 (27.7%)	18 (13.1%)		6 (22.2%)	10 (21.7%)	
**MP 3**	23 (63.9%)	76 (55.5%)		18 (66.7%)	26 (56.5%)	
**MP 4**	2 (5.6%)	23 (16.8%)		2 (7.4%)	5 (10.9%)	
**MP 5**	1 (2.8%)	18 (13.1%)		1 (3.7%)	4 (8.7%)	
**Well-responsive^b^**	3 (8.4%)	41 (29.9%)	**<0.0001**	3 (11.1%)	9 (19.6%)	0.539
**bpCR^c^**	1 (2.8%)	18 (13.1%)	0.142	1 (3.7%)	4 (8.7%)	0.737

^**a**^NCT, neoadjuvant chemotherapy, ^**b**^Well-responsive, include MP 4-5, ^**c**^bpCR, pathological complete response in breast. Bold values are statistically significant.

Baseline and post-treatment Ki-67 levels in the NET group were significantly lower than those observed in the NCT group (*p* < 0.001). Although both groups demonstrated a comparable reduction in Ki-67 levels post-treatment (*p* = 0.127), a greater proportion of patients in the NET group (94.4%) achieved Ki-67 levels ≤ 10% after 4–6 months of treatment, in contrast to only 56.9% in the NCT group (*p*<0.0001) (**Table 6A**). In both cohorts, patients with higher baseline Ki-67 levels (*p* < 0.05) exhibited a significant decrease in Ki-67 (*p* < 0.05) within the ‘well-responsive ‘cohort (MP 4-5) compared to those in the MP 1–3 group (**Table 6B**). However, post-treatment Ki-67 levels did not reveal significant differences between the well-responsive cohort (MP 4-5) and the MP 1–3 group in either treatment group (**Table 6B**).

**Table 6A:** Status of Ki-67 before and after PSM in both groups.

Before PSM	Dalpiciclib+AI (36)	NCT^a^ (137)	*P*
**Baseline**	15.0 (8.5, 20.0)	30.0 (15.0, 40.0)	**<0.001**
**Post surgery**	2.0 (1.3, 10.0)	10.0 (3.0, 20.0)	**<0.001**
**Range of changes**	9.0 (3.0, 14.8)	12.0 (2.5, 28.0)	0.127
**Post surgery ≤ 10%, n(%)**	34 (94.4%)	78 (56.9%)	**<0.0001**
After PSM	Dalpiciclib+AI (27)	NCT^a^ (46)	*P*
**Baseline**	15.0 (10.0, 20.0)	20.0(10.0, 22.5)	0.456
**Post surgery**	2.0 (1.0, 10.0)	10.0 (5.0, 20.0)	**<0.001**
**Range of changes**	10.0 (3.0, 15.0)	5.0 (0.0, 15.5)	0.185
**Post surgery ≤ 10%, n(%)**	26 (96.3%)	28 (60.9%)	**0.001**

^**a**^NCT, neoadjuvant chemotherapy. Bold values are statistically significant.

**Table 6B:** Status of Ki-67 before and after PSM in ‘MP 4-5’ (Well- responsive) and ‘MP 1-3’ groups.

Before PSM	Dalpiciclib+AI			NCT^a^		
	MP 1-3 (33)	MP 4-5 (3)	*P*	MP 1-3 (96)	MP 4-5 (41)	*P*
**Baseline**	10.0 (8.0, 20.0)	30.0 (20.0, -)	**0.027**	20.0 (10.0, 40.0)	40.0 (20.0, 60.0)	**0.001**
**Post surgery**	2.0 (1.25,10.0)	3.0 (1.0, -)	0.977	6.5 (3.0, 20.0)	15.0 (3.0, 30.0)	0.073
**Range of changes**	8.0 (3.0, 14.0)	27.0 (10.00, -)	**0.041**	10.0 (0.0, 21.5)	18.0 (8.5, 30.0)	**0.015**
After PSM	Dalpiciclib+AI			NCT^a^		
	MP 1-3 (24)	MP 4-5 (3)	*P*	MP 1-3 (37)	MP 4-5 (9)	*P*
**Baseline**	15.0 (6.3, 20.0)	30.0 (20.0, -)	**0.042**	15.0 (10.0, 20.0)	20.0 (20.0, 40.0)	**0.018**
**Post surgery**	2.0 (1.2, 10.0)	3.0 (1.0, -)	0.968	8.0 (5.0, 17.5)	15.0 (3.0, 30.0)	0.413
**Range of changes**	9.5 (3.0, 14.0)	27.0 (10.0, -)	0.063	5.0 (0.0, 13.5)	15.0 (3.0, 30.0)	0.231

^**a**^NCT, neoadjuvant chemotherapy. Bold values are statistically significant.

In the NET setting, two patients (5.6%) achieved a PEPI score of 0, while the remaining patients (94.4%) had a PEPI score of ≥ 1 (**Table 7**). Notably, neither of the two patients with a PEPI score of 0 attained a pCR in the breast or lymph nodes; both were classified with a grade of MP 2 and an RCB score of 2.

**Table 7:** Status of preoperative endocrine prognostic index (PEPI) before and after PSM in Dalpiciclib+AI group.

	Before PSM (36)	After PSM (27)
PEPI 0, n(%)	2 (5.6%)	1 (3.7%)
PEPI 1, n(%)	1 (2.8%)	0 (0.0%)
PEPI 2, n(%)	0 (0.0%)	0 (0.0%)
PEPI 3, n(%)	16 (44.4%)	12 (44.45%)
PEPI 4, n(%)	14 (38.8%)	12 (44.45%)
PEPI 5, n(%)	2 (5.6%)	1 (3.7%)
PEPI 6, n(%)	1 (2.8%)	1 (3.7%)

#### After PSM

**Figure 1:**
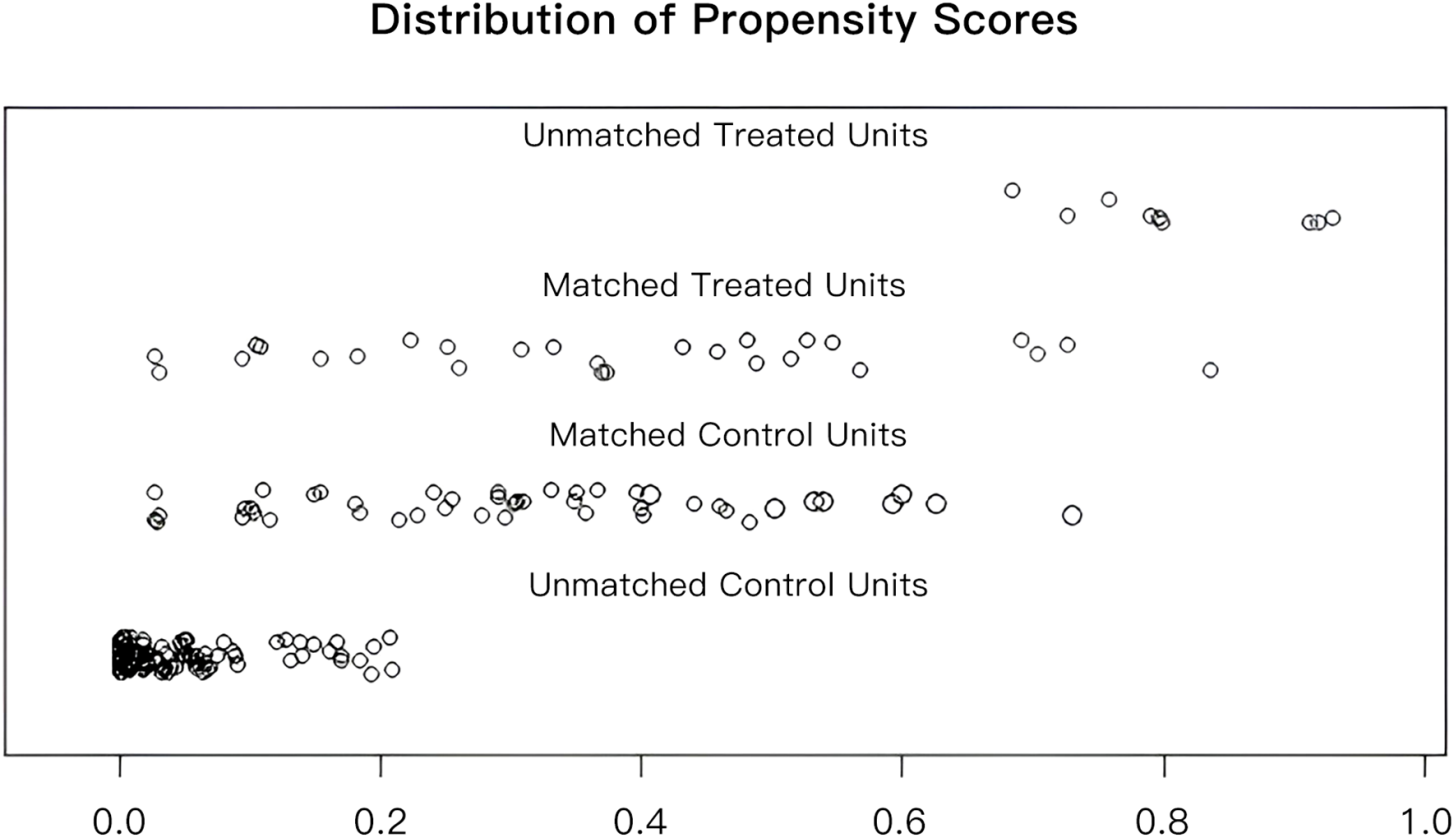
Propensity score scatter plot before and after the PSM matching.

**Figure 2:**
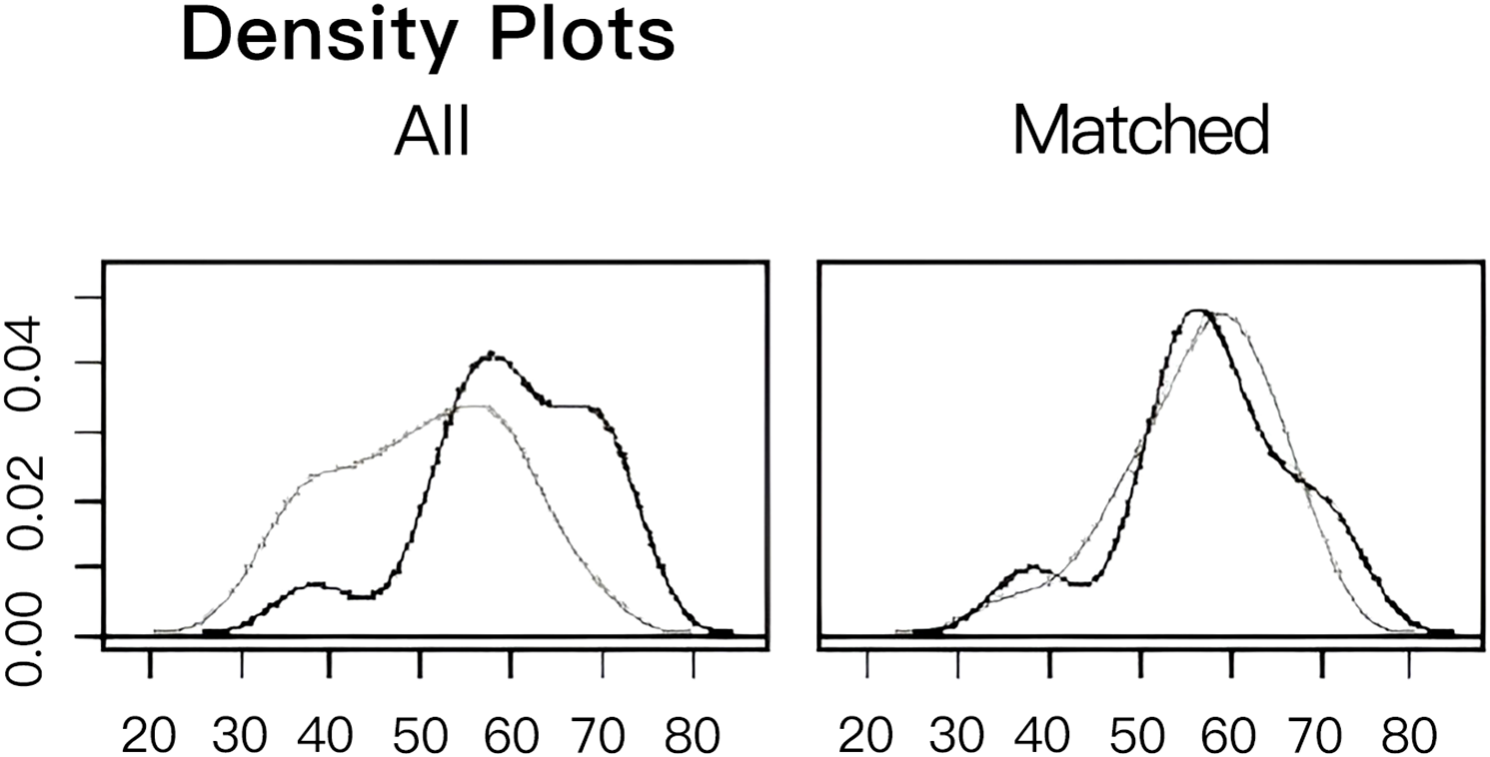
Distribution histogram of standard deviation.

**Figure 3:**
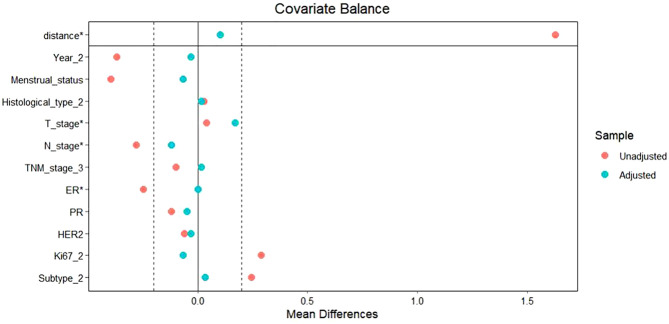
Validation of PSM using SMDs.

**Figure 4:**
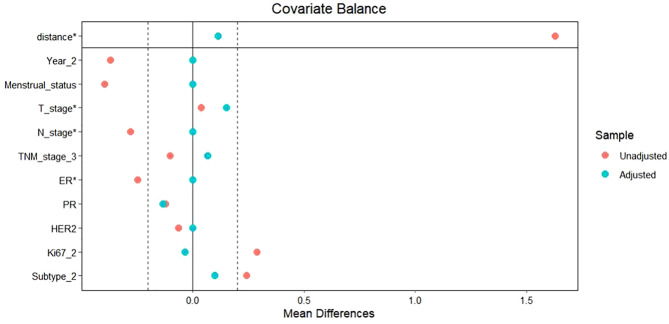
Sensitivity analyses for PSM.

To facilitate a precise comparison between NET and NCT, cases were matched based on clinicopathological characteristics at a ratio of 1:2, employing a caliper value of 0.5. Post-matching analysis revealed a more consistent distribution of propensity scores between the two groups (**Figure 1**), with the standard deviation concentrated around zero (**Figure 2**). The NET group comprised 27 patients, while the NCT group included 46 patients. Tumor characteristics were well-balanced between the two groups (**Table 8**), indicating a convergence of baseline conditions. The results of validating PSM using SMDs are presented in **Table 9** and **Figure 3**. Further sensitivity analyses were conducted to assess the robustness of the results. In this study, the covariates used for PSM included Year, menstrual status, histological type, T stage, N stage, TNM stage, ER, PR, HER2, Ki-67, and subtype. The initial matching resulted in 27 patients in the dalpiciclib + AI group and 46 patients in the NCT group, with all SMDs being within 0.2 (**Table 10**, **Figure 4**). After the exclusion of histological type, PSM was performed again. This yielded 30 patients in the dalpiciclib + AI group and 33 patients in the NCT group, with all SMDs still within 0.2, which indicated that the PSM in this study passed the sensitivity test and the model was relatively stable.

**Table 8:** Characteristics of patients after PSM in both groups.

	Dalpiciclib+AI (27)	NCT^a^ (46)	*P*
Median age	57	57.5	0.595
Age, n (%)			0.288
≤ 50	2 (7.4%)	9 (19.6%)	
>50	25 (92.6%)	37 (80.4%)	
Menstrual status, n (%)			0.548
premenopausal	7 (25.9%)	15 (32.6%)	
postmenopausal	20 (74.1%)	31 (67.4%)	
Histological type, n (%)			0.722
IDC^b^	24 (88.9%)	41 (89.1%)	
others	3 (11.1%)	5 (10.9%)	
T stage, n (%)			0.532
T1	10 (37.1%)	13 (28.3%)	
T2	12 (44.4%)	26 (56.5%)	
T3	3 (11.1%)	2 (4.3%)	
T4	2 (7.4%)	5 (10.9%)	
N stage, n (%)			0.905
N0	1(3.7%)	3 (6.5%)	
N1	22 (81.5%)	38 (82.7%)	
N2	2 (7.4%)	3 (6.5%)	
N3	2 (7.4%)	2 (4.3%)	
TNM stage^c^, n (%)			0.45
II	19 (70.4%)	36 (78.3%)	
III	8 (29.6%)	10 (21.7%)	
ER^d^, n (%)			1.0
1-10%	0 (0%)	0 (0%)	
11-50%	0 (0%)	0 (0%)	
> 50%	27 (100%)	46 (100%)	
PR^e^ n (%)			0.942
Positive	24 (88.9%)	42 (91.3%)	
Negative	3 (11.1%)	4 (8.7%)	
HER2^f^, n (%)			0.468
0-expression	4 (14.8%)	10 (21.7%)	
Low-expression	23 (85.2%)	36 (78.3%)	
Ki-67, n (%)			0.889
≤ 30%	24 (88.9%)	39 (84.8%)	
>30%	3 (11.1%)	7 (15.2%)	
Subtype, n (%)			0.743
Luminal A	8 (29.6%)	12 (26.1%)	
Luminal B	19 (70.4%)	34 (73.9%)	

^**a**^NCT, neoadjuvant chemotherapy, ^**b**^invasive ductal carcinoma, ^**c**^based on the 8th edition of the American Joint Committee on Cancer (AJCC), ^**d**^ER, estrogen receptor, ^**e**^PR, progesterone receptor, ^**f**^HER2, human epidermal growth factor receptor 2.

**Table 9:** Validation of PSM using SMDs.

Variables	SMDs	Threshold
Year	-0.033	<0.2
Menstrual_status	-0.067	<0.2
Histological_type	0.017	<0.2
T stage	0.170	<0.2
N stage	-0.120	<0.2
TNM stage	0.017	<0.2
ER^a^	0.000	<0.2
PR^b^	-0.050	<0.2
HER2^c^	-0.033	<0.2
Ki-67	-0.067	<0.2
Subtype	0.033	<0.2

^**a**^ER, estrogen receptor, ^**a**^PR, progesterone receptor, ^**c**^HER2, human epidermal growth factor receptor 2.

**Table 10:** Sensitivity analyses for PSM.

Variables	SMDs	Threshold
Year	<0.001	<0.2
Menstrual status	<0.001	<0.2
Histological type	0.1507	<0.2
T stage	<0.001	<0.2
N stage	0.0667	<0.2
TNM stage	<0.001	<0.2
ER^a^	-0.1333	<0.2
PR^b^	<0.001	<0.2
HER2^c^	-0.0333	<0.2
Ki-67	0.1	<0.2
Subtype	<0.001	<0.2

^**a**^ER, estrogen receptor, ^**b**^PR, progesterone receptor, ^**c**^HER2, human epidermal growth factor receptor 2.

After the PSM, no significant difference in clinical response, as assessed through imaging evaluation, was observed between the two groups (ORR: NET 62.9% *vs.* NCT 45.7%, *p* = 0.153, **Table 3**).

Following matching, the proportions of RCB in the NET cohort were observed as follows: 0% for RCB 0, 0% for RCB 1, 48.2% for RCB 2, and 51.8% for RCB 3. In contrast, the NCT group exhibited proportions of 6.5% for RCB 0, 10.9% for RCB 1, 28.3% for RCB 2, and 54.3% for RCB 3. The incidence of pCR in both breast tissue and lymph nodes (RCB 0) was comparable between the NET and NCT groups, with rates of 0% and 6.5%, respectively (*p* = 0.457, **Table 4**). Additionally, no significant differences were noted between the groups regarding the rates of bpCR (NET vs. NCT: 3.7% vs. 8.7%, *p* = 0.737, **Table 5**) or the ‘well-responsive’ status (NET vs. NCT: 11.1% vs. 19.6%, *p* = 0.539, **Table 5**).

Post-treatment Ki-67 levels in the NET group remained significantly lower than those observed in the NCT group, as indicated prior to matching (*p* < 0.001) (**Table 6A**). Both groups demonstrated a comparable reduction in Ki-67 levels following treatment (*p*=0.185) (**Table 6A**). Consistent with the pre-matching findings, a greater proportion of patients (96.3%) achieved Ki-67 levels ≤ 10% following NET compared to those receiving NCT (96.3% vs. 60.9%, *p* = 0.001) (**Table 6A**). Additionally, a higher percentage of patients in both groups presented with elevated baseline Ki-67 levels (*p* > 0.05) (**Table 6B**).

Similarly, after matching, only one patient (3.7%) achieved a PEPI 0 through NET (**Table 7**), with an MP grading of 2 and RCB 2.

### Safety

#### Before PSM

The most frequently reported adverse events (AEs) of any grade in the NET group included leucopenia (97.2%), neutropenia (97.2%), anemia (66.7%), and thrombocytopenia (55.6%). In contrast, the NCT group exhibited a higher prevalence of AEs, with leucopenia (100%), neutropenia (100%), gastrointestinal disorders (93.4%) (including mucositis, dysgeusia, diarrhea, constipation, nausea, and vomiting), peripheral neuropathy (90.5%), anemia (89.1%), alopecia (89.1%), and thrombocytopenia (75.2%). Detailed information regarding AEs is provided in **Table 11**. Statistical analysis revealed that the incidences of leucopenia and neutropenia were comparable between the two groups (*p* < 0.05); however, anemia (*p* = 0.001), thrombocytopenia (*p* = 0.021), gastrointestinal disorders (*p* < 0.0001), peripheral neuropathy (*p* < 0.0001), and alopecia (*p* < 0.0001) were significantly more frequent in the NCT group. Febrile neutropenia (FN) was reported in 25 patients (18.2%) within the NCT group, whereas no cases were documented in the NET group (*p* = 0.006). Grade 3–4 toxicities were predominantly hematologic in both cohorts (**Table 11**). The incidence of leucopenia (*p* = 0.007), neutropenia (*p* = 0.007), and anemia (*p* = 0.015) was significantly lower in patients receiving NET compared to those treated with NCT.

**Table 11:** Adverse events before and after PSM in both groups.

	Before PSM				After PSM			
	Dalpiciclib+AI (36)		NCT^a^ (137)		Dalpiciclib+AI (27)		NCT^a^ (46)	
	Any grade	Grade 3-4	Any grade	Grade 3-4	Any grade	Grade 3-4	Any grade	Grade 3-4
Leucopenia	35 (97.2%)	12 (33.3%)	137 (100%)	80 (58.4%)	26 (96.3%)	11 (40.7%)	46 (100%)	31 (67.4%)
Neutropenia	35 (97.2%)	13 (36.1%)	137 (100%)	84 (61.3%)	26 (96.3%)	11 (40.7%)	46 (100%)	31 (67.4%)
Anaemia	24 (66.7%)	3 (8.3%)	122 (89.1%)	38 (27.7%)	18 (66.7%)	3 (11.1%)	43 (93.5%)	12 (26.1%)
Thrombocytopenia	20 (55.6%)	2 (5.6%)	103 (75.2%)	26 (19.0%)	16 (59.3%)	2 (7.4%)	38 (82.6%)	9 (19.6%)
Gastrointestinal disorders	7 (19.4%)	0	128 (93.4%)	0	7 (25.9%)	0	44 (95.7%)	0
Febrile neutropenia	0	0	25 (18.2%)	0	0	0	8 (17.4%)	0
Alopecia	3 (8.3%)	0	122 (89.1%)	0	2 (7.4%)	0	41 (89.1%)	0
Peripheral neuropathy	0	0	124 (90.5%)	0	0	0	39 (84.8%)	0

^**a**^NCT, neoadjuvant chemotherapy.

#### After PSM

The AEs following matching are summarized in **Table 11**. The incidence of leucopenia (*p* = 0.37) and neutropenia (*p* = 0.37) was comparable between the groups post-matching. However, the NCT group exhibited significantly higher frequencies of anemia (*p* = 0.008), thrombocytopenia (*p* = 0.028), gastrointestinal disorders (*p* < 0.0001), peripheral neuropathy (*p* < 0.0001), and alopecia (*p* < 0.0001). The occurrence rate of FN was also higher in the NCT group(17.4%vs.0%), although this difference did not achieve statistical significance (*p* = 0.056). Regarding grade 3–4 AEs, leucopenia and neutropenia were more prevalent in the NCT cohort (*p* < 0.05), while the rates of anemia and thrombocytopenia were relatively similar (*p* > 0.05) (**Table 11**).

## Discussion

NCT remains the standard of care for locally advanced breast cancer (LABC). NCT offers significant benefits, particularly in patients with HER2-positive and triple-negative breast cancer (TNBC), achieving a pCR rate of 50%-60% (31–33) and correlating with favorable long-term survival outcomes in populations that attain pCR (34, 35). In contrast, due to lower chemotherapy sensitivity (36), pCR rates in luminal tumors are less than 20% (37). For these patients, NET emerges as a viable alternative to NCT. NET has been extensively evaluated in clinical trials (37), however, pCR rates remain limited compared to NCT (1, 3), even with the incorporation of CDK4/6i such as ribociclib or palbociclib (16, 38). Consequently, given the current insufficient and conflicting evidence, several critical questions necessitate further exploration: 1) What is the role of ET in the neoadjuvant setting for HR+ HER2 negative patients? 2) Does the introduction of CDK4/6i confer an advantage in pCR or survival compared to NCT? 3) If so, how can we optimize the patient populations suitable for CDK4/6i-based NET? 4) What are the optimal endpoints and duration for NET? In this context, we investigated the efficacy and safety of dalpiciclib in combination with an AI compared to NCT in patients with HR+ HER2 negative breast tumors, aiming to provide additional evidence to address the aforementioned questions. To our knowledge, this study represents the first comparison between dalpiciclib-based NET and NCT.

Considering the aggressiveness observed in premenopausal individuals, the majority of studies have focused on postmenopausal cohorts (37). Consistent with previous findings, our study revealed that NET was more frequently administered to women over the age of 50 years (*p* < 0.0001) and to postmenopausal populations (*p* < 0.0001). Notably, we also treated a subset of premenopausal patients (17.4%) with NET, given the enhanced efficacy observed when combined with OFS (1, 7, 39). Importantly, none of these premenopausal patients experienced disease progression (PD), indicating the potential feasibility of dalpiciclib-based NET in this demographic, which aligns with the findings reported by Iwamoto et al. (40). Furthermore, compared to those receiving NCT, a greater proportion of patients with Ki-67 levels ranging from 1-30% and classified as Luminal A subtype underwent NET (*p* < 0.05), reflecting the consideration of their heightened sensitivity to ET.

In light of the significant role of dalpiciclib in metastatic breast cancer (MBC), we investigated its potential in the NAT setting. We administered 4 to 6 months of dalpiciclib-based NET, drawing on treatment cycles from previous neoadjuvant trials involving CDK4/6is (15–18, 38). However, our analysis did not reveal a superiority in clinical and pathological responses of NET compared to NCT, even after PSM, which aligns with findings from the only two studies (NeoPAL and CORALEEN) that have compared CDK4/6i-based NET with chemotherapy (16, 38). The NeoPAL trial (16) established RCB and pCR in the breast as primary endpoints, reporting RCB 0–1 rates of 7.7% (RCB 0: 3.8%, RCB 1: 3.8%) for the letrozole-palbociclib arm versus 15.7% (RCB 0: 5.9%, RCB 1: 9.8%) for the chemotherapy arm, with pCR rates of 3.8% and 5.9%, respectively. In the CORALLEEN trial (38), comparing ribociclib plus letrozole and NCT, the rate of RCB 0 was 0% vs 5.8%, and ORR was 57.2% vs 78.8%. Furthermore, prior evidence suggests that the addition of CDK4/6is does not enhance the pathological response compared to ET alone (4–7, 17). One potential explanation for this unexpected outcome is the insufficient duration of NET, as extended treatment duration has been associated with improved clinical responses and a higher incidence of BCS (37, 41). Notably, NET has been shown to yield the most significant tumor shrinkage between 6 months and 1 year from the initiation of therapy (42). Additionally, RCB and pCR may not serve as optimal endpoints for evaluating response in the context of NET; therefore, we also assessed changes in Ki-67 and the PEPI scores following NAT.

Evidence indicates that a decrease in Ki-67 after a short-term treatment correlates significantly with long-term survival outcomes (2). Consequently, the changes in on-treatment Ki-67 levels after 2–4 weeks of NET were investigated as a potential surrogate marker for NET efficacy (37). In the Z1031B trial, patients were classified as non-responsive to NET if their Ki-67 levels remained above 10% after 2–4 weeks of treatment (43). However, due to the inherent limitations of on-treatment biopsies, we assessed Ki-67 status both prior to NAT and post-surgery, observing significant reductions in both treatment groups. Although Ki-67 levels ≤ 10% were more frequently noted post-treatment in patients receiving NET, this did not well correlate with clinical (ORR) or pathological (RCB 0 and bpCR) well-responsiveness. Furthermore, we found that patients with higher baseline Ki-67 levels demonstrated a greater likelihood of well-responsiveness (MP 4-5) to both treatment modalities (*P*<0.05). However, we did not observe any significant impact of post-treatment Ki-67 levels or changes in Ki-67 on well-responsiveness. Liebscher SC et al. (44) also reported that a decrease in Ki-67 at day 14 was not predictive of response as assessed by ultrasound or residual tumor bed cellularity (RTBC). This finding contrasts with the conclusions drawn from the PALLET trial (17). Therefore, the role of Ki-67 in the context of NET remains complex and warrants further investigation.

The PEPI score, which is associated with a favorable prognosis, is widely utilized as a prognostic index following NET. It incorporates several prognostic factors, including ER status, pathological tumor size, nodal involvement, and Ki-67 expression (43). Previous studies, including the P024 and IMPACT trials (27), have demonstrated a significant association between the PEPI score and relapse-free survival (RFS) in patients undergoing NET. In our study, patients who achieved a PEPI score of 0 through NET, both before and after matching, did not attain a bpCR or a RCB of 0. Similarly, a prospective, multi-center, non-randomized controlled trial reported that 36.4% of patients achieved a PEPI score of 0, yet the rate of pCR (ypT0/is ypN0) was only 2.5% following NET with letrozole alone (45). The CORALLEEN trial indicated that 22.4% of patients achieved a PEPI score of 0 with ribociclib and letrozole treatment; however, the rate of pathological complete response (ypT0/is ypN0) or RCB 0 was 0% (38). These findings suggest that the PEPI score may not correlate well with pCR or RCB, indicating that these endpoints may not be optimal for evaluating the efficacy of NET. Considering the relationship with long-term survival, PEPI may potentially serve as an efficacy evaluation index for NET. It is worth mentioning that in our study, only 2 individuals (5.6%) had a PEPI score of 0, which may be related to the small sample size. Moreover, this project is not prospective randomized controlled, and the efficacy may be influenced by several factors. Therefore, large-scale prospective studies are needed to further validate the role of PEPI in dalpiciclib-based NET.

There is currently no consensus regarding the potential of NET to enhance the BCS rate, as evidenced by conflicting results in the literature. In our study, the BCS rates in the two therapeutic groups were comparable both prior to and following matching (*p* > 0.05), which is consistent with the findings of the NeoPAL trial (16). Conversely, the majority of previous studies have reported a significant increase in BCS rates among patients receiving NET compared to those undergoing NCT (1, 3). This discrepancy may be attributed to variations in sample sizes and the associated medical backgrounds of the study populations.

Overall, our findings indicate that the combination of dalpiciclib and AI demonstrates a more favorable safety profile compared to NCT, particularly concerning hematological and non-hematological adverse events, including febrile neutropenia, gastrointestinal reactions, alopecia, and neurotoxicity. These results align with conclusions drawn from prior studies (16, 38). As this is a retrospective study, we were unable to collect sufficient data on AEs, and we only reported several AEs with relatively high incidence. Based on available data from other studies in NET setting, the proportion of AEs of grade 3 or higher in the combination of palbociclib and letrozole for NET was 39.6% (21/53) (16). The most common all-grade adverse events were diarrhea (62%), constipation (44%), and nausea (42%) in abemaciclib plus anastrozole (15), while the most common grade 3–4 AEs in ribociclib plus letrozole were neutropenia (43%) and elevated alanine aminotransferase concentrations (20%) (38). Hematological toxicity is the major adverse reaction shared by all CDK4/6is, however, there are still differences in the AEs profiles of different CDK4/6is. Although there are few direct comparisons of AEs among these drugs, previous data suggest that dalpiciclib is associated with relatively minor hepatotoxicity (25, 26).

Current evidence suggests that CDK4/6i-based NET may serve as viable candidates for NCT due to their comparable efficacy and improved safety profile. However, the optimal therapeutic regimens following NET remain uncertain. A large-scale trial employing multigene assays indicated that adjuvant CT is not recommended for postmenopausal patients with pathological N0–3 status and low to intermediate risk scores, as determined by the Oncotype Dx assay (46, 47). Furthermore, a phase III study demonstrated a significant improvement in disease-free survival (DFS) with the combination of CT and ET (CT+ET) compared to ET alone in populations with a risk score of 20% or higher (*p* = 0.026). Given the limited evidence available, further investigation into postoperative adjuvant therapeutic strategies following NET is warranted.

It should be mentioned that among the patients included in this study, the surgery was performed 1–4 weeks following NAT. In fact, almost all patients completed the surgery around 2 weeks. Only two patients who received NCT completed the surgery at the end of the first week and the beginning of the fourth week due to personal reasons, when their overall condition permitted. Considering that this timing would not have a negative impact on the patients, and both patients actually recovered well after surgery, we did not exclude these two patients.

To summarize, returning to the initial question: What is the role of NET for HR+ HER2 negative populations, even with CDK4/6is? Prat et al. (38) found that certain postmenopausal patients with high-risk Luminal B subtype and early-stage tumors could achieve molecular downstaging through treatment with ribociclib and letrozole. Cottu et al. (16) demonstrated that the combination of letrozole and palbociclib could serve as a viable alternative to NCT in early high-risk Luminal breast cancer. Additionally, several studies have been conducted to evaluate subsequent therapeutic regimens based on short-term changes in Ki-67 during a period of NET (18, 27, 48). Cao et al. (49) suggested that, despite higher response rates associated with NCT, NET could achieve both tumor (T) and nodal (N) downstaging, as well as pCR in breast or nodal areas. Furthermore, NET may facilitate the deescalation of surgical intervention when chemotherapy may be ineffective based on genomic testing or poorly tolerated. However, our data did not demonstrate any curative advantage of dalpiciclib-based NET over NCT. Nevertheless, given the comparable therapeutic responses and favorable safety profile, the combination of dalpiciclib and an AI may represent a potential alternative to NCT.

This study represents a preliminary investigation into the potential efficacy of dalpiciclib in NET. However, several limitations must be acknowledged. First, the study is a single-center retrospective analysis, which may limit the generalizability of the findings. Currently, a domestic multi-center phase II randomized clinical trial comparing dalpiciclib plus ET and NCT is ongoing in terms of patient enrollment. We expect that this study will provide more reliable and robust data. Second, as we all known breast cancer biology and treatment responses vary across populations, and potential ethnic or genetic also influences on drug efficacy. Due to drug availability, research on dalpiciclib involving foreign populations has not yet been initiated. It is hoped that international multi-center clinical trials will be conducted in the future to further evaluate the efficacy and safety of dalpiciclib combined with ET in NAT setting. Third, due to the limited sample size and considering the power of statistical analysis, we did not conduct subgroup analysis in the dalpiciclib group. The ongoing domestic multi-center phase II randomized controlled clinical trial will disclose data in this regard. Fourth, the adjuvant regimen administered post-surgery is not specified. Lastly, the follow-up duration is limited, and survival data are not available. We will further follow up on the survival status of these individuals in the future.

## Conclusions

This study represented a preliminary exploration of dalpiciclib in NET, and provides evidence suggesting that the combination of dalpiciclib and AI exhibits a comparable safety profile and therapeutic response to NCT in patients with HR+ HER2 negative breast tumors. Furthermore, we propose that RCB and pCR may not serve as optimal endpoints for evaluating the efficacy of CDK4/6i-based NET. PEPI score may be a better indicator, but further studies are needed to confirm it. It may even be necessary to explore new biomarkers to determine appropriate endpoints. To further elucidate and verify the implications of this treatment regimen in the neoadjuvant setting, data from large-scale, multi-center, prospective randomized controlled trial is warranted.

## Data Availability

The original contributions presented in the study are included in the article/supplementary material. Further inquiries can be directed to the corresponding author.
